# The PancreaSure Multianalyte Blood Test Performs Well at Distinguishing Early-Stage Pancreatic Ductal Adenocarcinoma from High-Risk Controls: A Retrospective Secondary Analysis

**DOI:** 10.3390/jcm15197355

**Published:** 2026-09-22

**Authors:** Tamas Gonda, Salvatore Paiella, Rosalie C. Sears, Erkut Borazanci, Patricio M. Polanco, Sonia S. Kupfer, Fay Kastrinos, Walter G. Park, Kelly Hedger, Lisa Ford, Thomas King, Norma A. Palma, Randall E. Brand

**Affiliations:** 1 Division of Gastroenterology & Hepatology, New York University, New York, NY 10016, USA; 2 Pancreatic Surgery Unit, Department of Surgery, Dentistry, Paediatrics and Gynaecology, University of Verona, 37134 Verona, Italy; 3 Department of Molecular and Medical Genetics, Brenden-Colson Center for Pancreatic Care, Knight Cancer Institute, Oregon Health & Science University, Portland, OR 97239, USA; 4 Department of Oncology, HonorHealth Research Institute, Scottsdale, AZ 85258, USA; 5 Division of Surgical Oncology, University of Texas Southwestern Medical Center, Dallas, TX 75390, USA; 6 Section of Gastroenterology, Hepatology and Nutrition, Department of Medicine, University of Chicago, Chicago, IL 60637, USA; 7 Department of Medicine, Division of Digestive and Liver Diseases, Columbia University Irving Medical Center, New York, NY 10032, USA; 8 Division of Gastroenterology and Hepatology, Stanford University, Palo Alto, CA 94305, USA; 9 Immunovia, Inc., Durham, NC 27713, USA; 10 Division of Gastroenterology, Hepatology and Nutrition, University of Pittsburgh Medical Center, Pittsburgh, PA 15213, USA

**Keywords:** pancreatic cancer, early-stage, early detection, diagnosis, blood-based biomarkers

## Abstract

**Background/Objectives**: Biomarkers that can facilitate early detection of pancreatic cancer and extend patient survival are a significant unmet need. PancreaSure is a serum biomarker signature that performed well at distinguishing Stage I and II pancreatic ductal adenocarcinoma (PDAC) from both high-risk and non-high-risk controls in two previous validations. The goal of this study was to perform a secondary analysis of test performance exclusively in individuals at high risk of PDAC due to a pathogenic variant in a susceptibility gene or strong family history of the disease. To this end, we extracted and analyzed raw data from appropriate samples used in the prior validations. **Methods**: Data from the two prior validations were pooled at the participant level, and sensitivity and specificity were estimated as pooled proportions with exact binomial 95% confidence intervals. One-sided exact binomial tests were used to compare the performance of the PancreaSure test to the performance goals of 65% sensitivity and 90% specificity. **Results**: PancreaSure detected early-stage PDAC with 77.5% sensitivity (95% CI, 66.0–86.5) and 93.6% specificity (95% CI, 91.8–95.1), significantly outperforming predefined success criteria. Test performance was consistent between Stage I (80.0% sensitivity) and Stage II (75.6% sensitivity) PDAC cases. PancreaSure also demonstrated favorable performance in individuals with only genetic susceptibility to PDAC (84.8% sensitivity, 94.5% specificity), with only a family history of the disease (79.2% sensitivity, 93.7% specificity), and with high-penetrance variants. **Conclusions**: The performance demonstrated by PancreaSure in genetic/familial high-risk individuals further suggests that it has the potential to facilitate early detection of PDAC in surveillance populations and warrants prospective evaluation as an adjunct to imaging.

## 1. Introduction

Pancreatic cancer is an aggressive malignancy that accounts for over 50,000 deaths each year and is one of the few cancers becoming more prevalent worldwide [1,2,3]. Pancreatic ductal adenocarcinoma (PDAC), which accounts for 90% of pancreatic cancers [4], has a 5-year survival rate of 3.1% when diagnosed at Stage IV [5], but improves to 44% when diagnosis occurs prior to metastasis and to 83% when diagnosed at Stage IA [5,6]. To facilitate early detection, yearly imaging surveillance is recommended for those at high risk of developing PDAC due to having a PGV in a PDAC susceptibility gene or a family history of PDAC [7,8,9]. Although routine imaging surveillance does increase early-stage diagnoses, late-stage and interval tumors still occur in surveillance populations, underscoring the need for complementary biomarker-based surveillance tests [10,11,12,13,14,15,16].

PancreaSure is a serum biomarker signature comprising tissue inhibitor of metalloproteinase 1 (TIMP1), intercellular adhesion molecule 1 (ICAM1), cathepsin D (CTSD), thrombospondin 1 (THBS1), and carbohydrate antigen 19-9 (CA 19-9) that was developed for the detection of Stage I and II PDAC in high-risk individuals undergoing surveillance [17]. In its first clinical validation, CLARITI, PancreaSure was tested in a cohort containing sporadic and non-sporadic Stage I and II PDAC cases (*n* = 202) and a control group that included individuals with genetic/familial susceptibility to PDAC (*n* = 730), as well as PDAC-negative individuals without genetic or familial risk (*n* = 156). In CLARITI PancreaSure distinguished Stage I and II PDAC from controls with 78.2% sensitivity and 93.5% specificity [18]. In a second independent validation, VERIFI, the test demonstrated 76.5% sensitivity and 87.8% specificity in a cohort of Stage I and II sporadic and non-sporadic PDAC cases (*n* = 115) and controls at high risk of PDAC due to genetic/familial susceptibility and/or the presence of mucinous pancreatic cysts (*n* = 270) [19].

The goal of this study was to perform a retrospective, secondary analysis of data exclusively from genetic/familial high-risk individuals. Due to the rarity of PDAC samples that capture Stage I and II disease in high-risk physiologies, validation cohorts must often include sporadic cases in addition to those with genetic/familial susceptibility to achieve appropriate statistical power. We overcame this challenge by extracting and analyzing raw data from a pool of appropriate samples that had been utilized in the CLARITI and VERIFI studies.

## 2. Materials and Methods

### 2.1. Study Endpoints

The primary study endpoint was to evaluate PancreaSure’s sensitivity and specificity against the predefined success criteria of 65% and 90%, respectively. Here, 65% sensitivity was chosen based on the sensitivity of CA 19-9 alone at detecting Stage I and II PDAC [17]. The performance of CA 19-9 is clinically relevant due to its status as one of the only biomarkers approved for use in PDAC patients and its wide use in pancreatic oncology [20,21]. Moreover, 90% specificity was established as a performance metric based on acceptable levels of specificity for early detection of rare cancers [22]. PDAC qualifies as a rare cancer due to its incidence rate of 1.5% in high-risk populations [10]. In the absence of test performance guidelines specifically for PDAC, 90% specificity is an appropriate benchmark for PancreaSure.

The secondary study endpoints were to evaluate test performance in Stage I cases alone and Stage II cases alone, as well as in clinically relevant subgroups, which included those with only genetic or only familial susceptibility to PDAC, and those carrying pathogenic variants in the *BRCA1/2* or *CDKN2A* genes.

### 2.2. Study Design and Population

The CLARITI and VERIFI validations were both retrospective and conducted according to the approved study protocols [18,19]. All study sites had Institutional Review Board approval for collection of blood samples and clinical data. All study participants provided written informed consent to the respective institutions that permitted use of their samples in either study. Samples that were eligible for the previous validations were chosen and deidentified as described [18,19]. Samples from which raw data were extracted and analyzed for the current study were selected from the combined pool of CLARITI and VERIFI samples. They included treatment-naive individuals recently diagnosed with Stage I or II PDAC who met genetic/familial high-risk criteria (*n* = 71) [18,19], as well as genetic/familial high-risk controls (*n* = 922). Sporadic cases, non-high-risk controls, and controls that were considered high-risk based on the presence of pancreatic cysts alone were not included. All participants represented in this study’s pooled cohort were enrolled via surveillance at the time of sample collection. PDAC diagnosis was confirmed by either EUS or MRI/MRCP plus biopsy (see Supplementary Table S1 for tumor stage distribution). Controls were evaluated for PDAC using EUS and/or MRI/MRCP with imaging-based follow-up surveillance for a minimum of 6 months. Asymptomatic individuals, as well as individuals with chronic pancreatic abnormalities or acute symptoms concerning of PDAC were represented in this pooled genetic/familial high-risk study population (Supplementary Table S2).

### 2.3. Biomarker Measurements

In the prior validations, signature analytes were measured as previously described [23,24], logarithmically transformed, and multiplied by an established coefficient for each analyte, which was determined during assay development [17,25]. The sum of results for each sample was assigned a positive or negative result based on a prespecified proprietary cutoff score that was also ascertained during assay development. This cutoff score was fully locked for the CLARITI and VERIFI validations and for the analysis of this study’s pooled dataset [25].

### 2.4. Statistical Analyses

Raw data from the CLARITI and VERIFI validations were pooled, and all performance estimates were calculated from the combined cohort counts. Sensitivity, specificity, positive predictive value, negative predictive value, and the positive likelihood ratio are reported with exact 95% confidence intervals. Predictive values reflect the observed prevalence of PDAC in the pooled dataset. In the full genetic/familial high-risk dataset, sensitivity and specificity were compared to the predefined success criteria of 65% and 90% using one-sided exact binomial tests. For subgroup analyses, calculated estimates were compared using Fisher’s exact test. For all tests, *p*-values < 0.05 were considered significant. All statistical analyses were performed using R, version 4.3.1 [26].

## 3. Results

### 3.1. Study Participant Demographics

The demographics of the pooled study population are shown in Table 1. The mean age of all participants was 62.1 years (SD ± 8.9 years), with cases (67.0 ± 9.9 years) being significantly older than controls (61.7 ± 8.7 years) (*p* = 0.001). Cases and controls had similar splits between males and females (*p* = 0.249) and between those with genetic risk (*p* = 0.711). The control group included significantly more individuals with familial risk than cases (*p* < 0.001).

### 3.2. Clinical Performance

#### Overall Performance

In the full cohort, PancreaSure distinguished Stage I and II non-sporadic PDAC from genetic/familial high-risk controls with 77.5% sensitivity (95% CI, 66.0–86.5%) and 93.6% specificity, significantly outperforming the goals of 65% sensitivity (*p* = 0.016) and 90% specificity (*p* < 0.001) (Table 2). The test also performed consistently between Stage I and II cases, demonstrating sensitivities of 80.0% and 75.6%, respectively (Table 3).

### 3.3. Genetic vs. Familial Risk

Next, we evaluated PancreaSure’s performance in individuals with only familial risk or only genetic risk of PDAC. The test demonstrated 79.2% sensitivity and 93.7% specificity in those who only reported familial risk of PDAC, and 84.8% sensitivity and 94.5% specificity in those who only reported genetic risk (Table 4). The difference in test performance was not statistically significant (sensitivity, *p* = 0.727; specificity, *p* = 0.746). See Supplementary Table S3 for the type and distribution of pathogenic variants across cases and controls.

### 3.4. High-Penetrance Mutations

Finally, we evaluated the performance of PancreaSure in individuals carrying pathogenic *BRCA1/2* or *CDKN2A* variants, which are associated with some of the highest lifetime risks of developing PDAC [27,28]. In those with *BRCA1/2* mutations, PancreaSure distinguished Stage I and II PDAC from controls with 86.7% sensitivity (95% CI, 59.5–98.3%) and 93.5% specificity (95% CI, 89.6–96.3%), similar to the performance observed in the complete genetic high-risk group (Table 5). *CDKN2A* variant carriers were not represented among PDAC cases, owing to the genetic rarity of such mutations. Although the absence of these individuals prevented a sensitivity analysis, the test identified *CDKN2A*-carrying controls with 96.4% specificity (95% CI, 87.7–99.6).

## 4. Discussion

PancreaSure is intended to detect Stage I and II PDAC in high-risk individuals undergoing surveillance due to a documented PGV in a PDAC susceptibility gene or a family history of pancreatic cancer. The test was previously validated in two independent cohorts of Stage I and II PDAC cases and controls [18,19]. The goal of this study was to further establish the test’s performance in genetic/familial high-risk individuals by performing a retrospective secondary analysis of data from this group.

PancreaSure significantly outperformed predefined success criteria in the pooled dataset. In the CLARITI and VERIFI validation studies PancreaSure performed consistently well between sporadic PDAC cases and cases that developed in high-risk individuals [18,19], and the results of this study confirm the test’s high sensitivity in PDAC patients with genetic/familial risk. This pooled analysis also demonstrated similar test performance between Stage I and II cases, consistent with the prior validations. High performance in Stage I cases alone is a unique strength of PancreaSure because biomarkers that perform well in Stage I and II combined, but poorly in Stage I alone, are unlikely to impact survival since many individuals diagnosed at Stage II succumb to illness within five years [6,29]. For this reason, high performance in Stage I cases is particularly noteworthy. Another strength of the test is its promising performance in genetic/familial high-risk controls. Underrepresentation of these individuals in discovery and validation studies has been cited as a primary reason why biomarker panels designed to facilitate early detection have been largely unsuccessful in clinical trials [30,31]. The test’s consistent performance in high-risk surveillance populations across two independent validations and this pooled secondary analysis suggests that is has the potential to aid early-stage diagnosis as an adjunct to routine imaging-based surveillance.

In this study, test performance was consistent between those with only genetic and only familial risk, and performance in individuals carrying pathogenic *BRCA1/2* or *CDKN2A* variants was encouraging. Pathogenic germline *CDKN2A* variants, including deep deletions, are among the highest penetrance genetic risk factors for PDAC, increasing lifetime risk by approximately 12-fold [32,33,34]. Compared with individuals who develop PDAC sporadically, carriers of pathogenic *CDKN2A* variants often experience a more aggressive disease course characterized by faster tumor progression, lower rates of resectability, shorter overall survival, and higher incidence of interval cancer [35,36]. Additionally, those with a germline *CDKN2A* mutation have an elevated risk of developing other cancers, especially familial atypical multiple-mole melanoma, and guidelines recommend that these individuals undergo biannual examination for melanoma [37]. Mutations in *BRCA 1* increase the lifetime risk of PDAC by approximately 2-fold, and *BRCA 2* mutations increase lifetime risk by 3- to 10-fold [38,39]. Compared with individuals who develop sporadic PDAC, carriers of pathogenic *BRCA1/2* variants develop PDAC approximately 10 years earlier and have shorter overall survival following diagnosis [40,41,42]. They too are at elevated risk of developing other cancers and are recommended to initiate annual surveillance for breast, ovarian, and prostate cancer at 30 years of age [8].

Among *CDKN2A* variant carriers in this study, PancreaSure identified controls with 96.4% specificity. The test demonstrated promising performance in *BRCA1/2* variant carriers as well, distinguishing Stage I and II PDAC from controls with 86.7% sensitivity and 93.5% specificity. For individuals who initiate cancer surveillance at a young age and may undergo annual screening for multiple malignancies over several decades, minimizing false positive test results along with the associated costs, cancer worry, and unnecessary follow-up procedures is essential to making surveillance sustainable over a lifetime. The specificity demonstrated by PancreaSure in these genetic subgroups suggests that it may have the potential to limit false positive test results, which could be particularly beneficial to those who will undergo surveillance for decades.

Although this study’s findings are promising, we acknowledge its limitations. PPV and NPV were calculated based on the prevalence of PDAC in the pooled cohort, which was much higher than its incidence of ~1.5% in high-risk populations, and as such, these findings should be interpreted cautiously. Certain clinical information was not available for PDAC cases, including tumor size and resectability, and tumor grade was not reported for one Stage II case. There was also a female predominance in the pooled dataset, and study participants with PDAC were significantly older than control participants. The age gap between cases and controls may have implications for measured analyte levels. Circulating CTSD has been shown to decrease with normal aging [43], while TIMP1, ICAM1, THBS1, and CA 19-9 tend to increase with age and have been linked to age-related inflammation, cardiovascular remodeling, and maladaptive aging responses [44,45,46,47,48,49,50]. Although PancreaSure performed similarly in both the CLARITI and VERIFI validations where the VERIFI cohort was balanced for age and sex while CLARITI was not, additional studies with age-balanced cohorts are needed to fully evaluate the impact of patient age on test performance. Another limitation was the lack of *CDKN2A* variant carriers among PDAC cases, which prevented a sensitivity analysis in this subgroup. Due to this limitation, along with the relatively small number of PDAC cases among *BRCA1/2* variant carriers, findings in both of these genetic subgroups should be considered hypothesis-generating and exploratory. Prospective, longitudinal studies are needed to fully characterize PancreaSure’s performance in these genetic subgroups. We also acknowledge that since asymptomatic individuals and individuals with chronic pancreatic abnormalities and acute symptoms concerning of PDAC were represented in the pooled study population, potential differences in test performance between these groups are currently unknown. However, retrospective analyses to evaluate this are underway.

Altogether, this study provides further evidence that PancreaSure distinguishes Stage I and II PDAC from genetic/familial high-risk controls with high sensitivity and specificity, suggesting that the test could improve detection of early-stage PDAC in surveillance populations as an adjunct to imaging.

## Figures and Tables

**Table 1:** Demographics of the pooled dataset.

	Cases (*n* = 71)	Controls (*n* = 922)	Total (*n* = 993)	*p*-Value
Age, Years				
Mean (SD)	67.0 (±9.9)	61.7 (±8.7)	62.1 (±8.9)	0.001
Min, Max	47.0, 83.0	45.0, 88.0	45.0, 88.0	
Gender, *n* (%)				
Female	41 (57.7%)	597 (64.8%)	638 (64.2%)	0.249
Male	30 (42.3%)	325 (35.2%)	355 (35.8%)	
Documented PGV, *n* (%)				
Yes	41 (57.7%)	505 (54.8%)	546 (55.0%)	
No	30 (42.3%)	417 (45.2%)	447 (45.0%)	0.711
FDR Diagnosed with PDAC, *n* (%)				
Yes	32 (45.1%)	612 (66.4%)	644 (64.9%)	
No	39 (54.9%)	310 (33.6%)	349 (35.1%)	<0.001

FDR = first degree relative; PGV = pathogenic germline variant.

**Table 2:** Test performance in the full dataset.

	Sensitivity (95% CI) (*n*/*N*)	Specificity (95% CI) (*n*/*N*)	PPV (95% CI)	NPV (95% CI)	LR+ (95% CI)
PancreaSure	77.5% (66.0–86.5) (55/71)	93.6% (91.8–95.1) (863/922)	48.2% (38.8–57.8)	98.2% (97.1–99.0)	12.11 (9.18–15.97)
Performance Goal	65%	90%			
*p*-value	0.016	<0.001			

CI = confidence interval; PPV = positive predictive value; NPV = negative predictive value; LR+ = positive likelihood ratio.

**Table 3:** Test performance in Stage I and Stage II PDAC cases.

	**Sensitivity (95% CI) (*n*/*N*)**	**Specificity (95% CI) (*n*/*N*)**	**PPV (95% CI)**	**NPV (95% CI)**
Stage I cases	80.0% (61.4–92.3) (24/30)	93.6% (91.8–95.1) (863/922)	28.9% (19.5–39.9)	99.3% (98.5–99.7)
Stage II cases	75.6% (59.7–87.6) (31/41)		34.4% (24.7–45.2)	98.9% (97.9–99.4)

CI = confidence interval; PPV = positive predictive value; NPV = negative predictive value.

**Table 4:** Performance in high-risk sub-groups.

Group	Cases	Controls	Sensitivity (95% CI) (*n*/*N*)	Sensitivity *p*-Value	Specificity (95% CI) (*n*/*N*)	Specificity *p*-Value
Diagnosed FDR only	24	398	79.2% (57.8–92.9) (19/24)	0.727	93.7% (90.9–95.9) 373/398	0.746
Documented PGV only	33	291	84.8% (68.1–94.9) (28/33)		94.5% (91.2–96.8) (275/291)	

CI = confidence interval; FDR = first degree relative; PGV = pathogenic germline variant.

**Table 5:** Performance in individuals with high-penetrance PGVs.

Group	Cases	Controls	Sensitivity (95% CI) (*n*/*N*)	Sensitivity *p*-Value ^1^	Specificity (95% CI) (*n*/*N*)	Specificity *p*-Value ^1^
Documented PGV	41	505	80.5% (65.1–91.2) (33/41)		93.5% (90.9–95.5) (472/505)	
*BRCA1/2* mutation	15	234	86.7% (59.5–98.3) (13/15)	0.713	93.5% (89.6–96.3) (219/234)	1.0
*CDKN2A* mutation	0	56	NA	NA	96.4% (87.7–99.6) (54/56)	0.563

^1^ Comparisons between genetic sub-groups and all individuals with a documented pathogenic germline variant (PGV). CI = confidence interval.

## Data Availability

The original contributions presented in this study are included in the article and Supplementary Materials. Further inquiries can be made to the corresponding author.

## Supplementary Materials

The following supporting information can be downloaded at https://www.mdpi.com/article/10.3390/jcm15197355/s1. Table S1: Tumor stage and CA 19-9 distribution; Table S2: Chronic pancreatic abnormalities and acute symptoms concerning for PDAC reported in the pooled study population; Table S3: Distribution of pathogenic germline variants by clinical status.

## Abbreviations

The following abbreviations are used in this manuscript:

CA 19-9	Carbohydrate antigen 19-9
CI	Confidence Interval
CTSD	Cathepsin D
FDR	First degree relative
ICAM1	Intercellular adhesion molecule 1
NPV	Negative predictive value
PDAC	Pancreatic ductal adenocarcinoma
PGV	Pathogenic germline variant
PPV	Positive predictive value
PtCh	Platinum-based chemotherapy
SD	Standard deviation
THBS1	Thrombospondin 1
TIMP1	Tissue inhibitor of metalloproteinase 1
