# Supplementary material for: The PancreaSure Multianalyte Blood Test Performs Well at Distinguishing Early-Stage Pancreatic Ductal Adenocarcinoma from High-Risk Controls: A Retrospective Secondary Analysis
Source: J Clin Med. 2026 Sep 22;15(19):7355. doi: 10.3390/jcm15197355 (PMC13666322; doi:10.3390/jcm15197355)
Supplement: Supplementary file 1 [file jcm-15-07355-s001.zip › jcm-4519873-supplementary.pdf]

Table S1. Tumor stage and CA 19-9 distribution.		
**PDAC Stage**	**n (%)**	**CA 19-9 (U/mL) mean (range)**
IA	9 (12.7%)	47.1 (0.6, 91.9)
IB	21 (29.6%)	277.1 (0.6, 1000)
IIA	10 (14.1%)	282.3 (3.5, 1000)
IIB	30 (42.3%)	294.8 (0.6, 1000)
II, grade unreported	1 (1.4%)	466.6

**Table S2.** Chronic pancreatic abnormalities and acute symptoms concerning for PDAC reported in the pooled study population.			
	Cases (n= 71)	Controls (n= 922)	Total (n= 993)
Hyperbilirubinemia (> 1.2 mg/dL), n (%)			
Yes	28 (39.4%)	2 (0.22%)	30 (3.02%)
No	32 (45.0%)	611 (66.3%)	643 (64.8%)
Not Reported	11 (15.5%)	309 (33.5%)	320 (32.3%)
Pancreatic Cysts, n (%)			
Yes	19 (26.8%)	328 (35.6%)	347 (35.0%)
No	52 (73.2%)	594 (64.4%)	646 (65.0%)
Not Reported	0 (0.0%)	0 (0.0%)	0 (0.0%)
Diabetes, n (%)			
Yes	28 (39.4%)	70 (7.6%)	98 (9.87%)
No	43 (60.6%)	851 (92.3%)	894 (90.0%)
Not Reported	0 (0.0%)	1 (0.11%)	1 (0.10%)
Jaundice, n (%)			
Yes	28 (39.4%)	1 (0.11%)	29 (2.92%)
No	43 (60.6%)	721 (78.2%)	764 (76.9%)
Not Reported	0 (0.0%)	200 (21.7%)	200 (20.1%)
Main Duct-Intraductal Papillary Mucinous Neoplasm (MD-IPMN)			
Yes	4 (5.6%)	7 (0.76%)	11 (1.11%)
No	42 (59.1%)	691 (75.0%)	733 (73.8%)
Not Reported	25 (35.2%)	224 (24.3%)	249 (25.1%)
Pancreatitis, n (%)			
Yes	10 (14.1%)	3 (0.33%)	13 (1.31%)
No	57 (80.3%)	918 (99.6%)	975 (98.2%)
Not Reported	4 (5.6%)	1 (0.11%)	5 (0.50%)
Unexplained Weight Loss > 10 lbs., n (%)			
Yes	32 (45.1%)	5 (0.54%)	37 (3.73%)
No	28 (39.4%)	656 (71.1%)	684 (68.9%)
Not Reported	11 (15.5%)	261 (28.3%)	272 (27.4%)

**Table S3**. **Distribution of pathogenic germline variants by clinical status**.			
**PGV**	**Cases (n= 43)**	**Controls (n= 519)**	**Total (n= 562)**
BRCA1	2	74	76
BRCA2	13	175	188
CDKN2A	0	55	55
ATM	7	82	89
PALB2	6	44	50
MSH6	0	8	8
MSH2	1	4	5
MLH1	0	3	3
Specific PGV not reported	14	74	88
Some study participants reported more than one pathogenic germline variant (PGV), which is why the total number of PGVs among controls outnumbers the total number of study participants in that group (n= 505).			
